# Transcriptional quiescence of paternal mtDNA in cyprinid fish embryos

**DOI:** 10.1038/srep28571

**Published:** 2016-06-23

**Authors:** Ming Wen, Liangyue Peng, Xinjiang Hu, Yuling Zhao, Shaojun Liu, Yunhan Hong

**Affiliations:** 1 State Ministry of Education Key Laboratory of Protein Chemistry and Developmental Biology, College of Life Sciences, Hunan Normal University, Changsha 410081, China; 2 Department of Biological Sciences, National University of Singapore, Singapore 117543, Singapore

## Abstract

Mitochondrial homoplasmy signifies the existence of identical copies of mitochondrial DNA (mtDNA) and is essential for normal development, as heteroplasmy causes abnormal development and diseases in human. Homoplasmy in many organisms is ensured by maternal mtDNA inheritance through either absence of paternal mtDNA delivery or early elimination of paternal mtDNA. However, whether paternal mtDNA is transcribed has remained unknown. Here we report that paternal mtDNA shows late elimination and transcriptional quiescence in cyprinid fishes. Paternal mtDNA was present in zygotes but absent in larvae and adult organs of goldfish and blunt-snout bream, demonstrating paternal mtDNA delivery and elimination for maternal mtDNA inheritance. Surprisingly, paternal mtDNA remained detectable up to the heartbeat stage, suggesting its late elimination leading to embryonic heteroplasmy up to advanced embryogenesis. Most importantly, we never detected the cytb RNA of paternal mtDNA at all stages when paternal mtDNA was easily detectable, which reveals that paternal mtDNA is transcriptionally quiescent and thus excludes its effect on the development of heteroplasmic embryos. Therefore, paternal mtDNA in cyprinids shows late elimination and transcriptional quiescence. Clearly, transcriptional quiescence of paternal mtDNA represents a new mechanism for maternal mtDNA inheritance and provides implications for treating mitochondrion-associated diseases by mitochondrial transfer or replacement.

The mitochondrion (MT) is a membraned organelle present in all eukaryotic organisms. MT converts the energy of food molecules into ATP to support cellular and organismal metabolism, and is involved also in regulating diverse processes such as apoptosis and innate immunity^1^,^2^. MT is a unique organelle in possessing a multicopy genome, namely mitochondrial DNA (mtDNA). The human mtDNA is a double-stranded circular molecule and 16,569 bp in length, has a D-loop as the control region for replication and transcription, and 37 genes for 13 proteins, 22 transfer RNAs and 2 ribosomal RNAs^3^. These mtDNA features are highly conserved in diverse animal phyla including fish^4^,^5^. Exceptions exist. For instance, medusozoan animals such as those in the genus Hydra have linear mtDNA molecules^6^, and the mytilid bivalve (*Musculista senhousia)* has mtDNA that show differences in size and gene number between male and female origins^7^. In addition, mtDNA of certain vertebrates such as fish may show size variations by the presence and copy number of repetitive sequences in the D-loop region^5^. In human, MT dysfunction and mtDNA mutation are causative for diseases such as diabetes mellitus and cancers^8^,^9^,^10^,^11^. Replacement of a mutant mtDNA by its wildtype version via pronuclear transfer has the potential to prevent transmission of mtDNA-associated diseases in primates including human^12^,^13^.

Many organisms are homoplasmic, because their cells possess a pool of homogeneous mtDNA molecules. Homoplasmy is very important for normal development, because heteroplasmy–mixing of even two different normal mtDNAs–may lead to genetic instability in mice^14^ and even human diseases^15^. One of the most important mechanisms to maintain homoplasmy is uniparental inheritance of mtDNA. Maternally uniparental inheritance (MUI) of mtDNA has been reported in a wide variety of organisms examined so far, including many invertebrates and all vertebrate species such as humans and other mammals^3^,^16^. Exceptions are certain bivalve mollusks, which show doubly uniparental inheritance (DUI)^17^. These mollusks have two distinct mtDNAs, namely female type (F-type) mtDNA and male type (M-type) mtDNA. The F- and M-type mtDNAs display more than 20% nucleotide sequence divergence. The transmission of two mtDNA types is, however, independent and uniparental, because the F-type mtDNA is transmitted through eggs to both female and male progeny, whereas the M-type mtDNA is transmitted through sperm to male progeny only. Consequently, female mollusks possess only F-type mtDNA and are thus hemoplasmic, and males are thus heteroplasmic because they have F-type in their somatic organs and M-type mtDNAs in their gonads. In these DUI organisms, M-type mtDNA plays an essential role in male sex determination, germline establishment, spermatogenesis and sperm function^18^,^19^,^20^.

Different degrees of paternal inheritance or leakage of mtDNA may occur even in organisms with demonstrated MUI such as Drosophila^21^,^22^. In human, paternal inheritance of mtDNA has been controversial. Paternal inheritance of mtDNA was suggested by linkage disequilibrium and recombination in mtDNA^23^. The best case of paternal inheritance of human mtDNA has been reported in a patient carrying a pathogenic mtDNA mutation^24^. Subsequent studies of patients with various mtDNA defects have, however, argued against paternal inheritance of human mtDNA^2^,^25^. Although M-type is transcribed in not only male germ cells^19^,^20^ but also the somatic cells^26^, it has remained unknown whether paternal mtDNA is transcribed in MUI organisms. In fish, we and others have reported MUI in medaka^27^ and recombination between maternal and paternal mtDNAs in hybrid triploids between goldfish and common carp^28^.

This study was aimed at investigation of the fate and behavior of paternal mtDNA in reciprocal hybrids between goldfish (*Carassius auratus* red var.) and blunt snout bream (*Megalobrama amblycephala*) as a model of cyprinid fishes. We show that MUI of mtDNA operates in both species by the elimination of paternal mtDNA during embryogenesis. Interestingly, we demonstrate that paternal mtDNA can persist to fairly advanced stages of embryogenesis and remains transcriptionally quiescent, excluding its phenotypic contribution to the developing embryos.

## Results

### Hybrid analysis system

**Figure 1:**
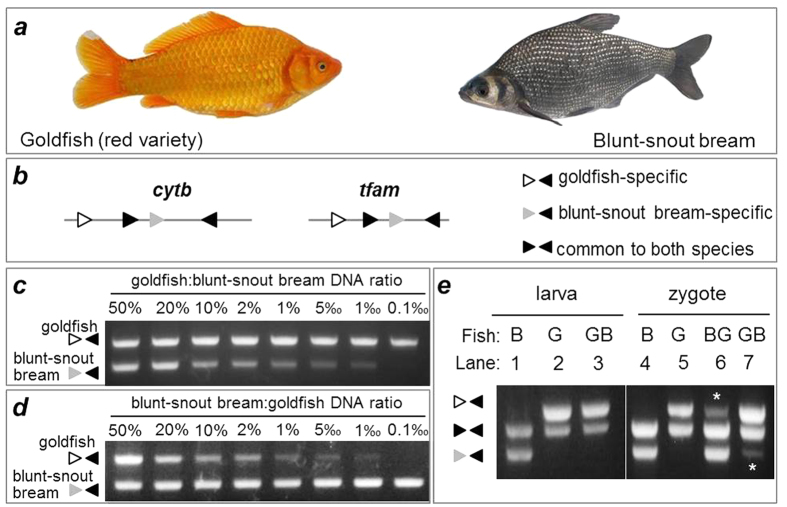
Origin and inheritance of mtDNA in cyprinid fishes. **(a**) Goldfish red variety and blunt-snout bream used for reciprocal hybridization. (**b**) Scheme of PCR primers for cytb and tfam, showing primers specific to goldfish (open arrowhead) or blunt-snout bream (grey arrowhead) and common to both species (black arrowheads). For more details see Figs S1 and S2. (**c**,**d**) Specificity and sensitivity of mtDNA detection by PCR. Genomic DNA mixtures between goldfish and the blunt-snout bream were prepared at various ratios and used for PCR analysis by using species-specific cytb primers. Notably, an amount as low as 1‰ is easily detectable. (**e**) PCR analysis of mtDNA origins, showing the absence of sperm cytb in the hybrid larvae between female goldfish and male blunt snout bream and the coexistence of maternal and paternal cytb in the zygotes from reciprocal hybridization. Asterisks depict sperm mtDNA. DNA was isolated from 20 pooled zygotes and embryos at each stage from parental species and reciprocal hybridization and analyzed by PCR at representative stages indicated. β-actin was used as a loading control. PCR and gels were run under the same conditions. B, blunt-snout bream; G, goldfish; BG, blunt-snout bream female × goldfish male; GB, goldfish female × blunt-snout bream male.

We make use of cyprinid hybrids as a model system to analyze the mtDNA behavior of different parental origins in developing embryos. Certain species of even distantly related taxa of the family Cyprinidae can easily be mated by artificial insemination procedures to produce hybrid embryos and even adults^29^. Examples are the goldfish (Fig. 1a; left panel) and blunt-snout bream (Fig. 1a; right panel), which belong to subfamilies Cyprininae and Cultrinae, respectively. Previously we have shown that the cross between female goldfish and male blunt-snout bream leads to the production of hybrid adult fish, whereas the embryos from the reciprocal cross, namely the cross between female blunt-snout bream and male goldfish, develop abnormally and die shortly after hatching^30^, indicating a possibility that nucleocytoplasmic incompatibility would play a key role in distant hybridization between these two species.

A pair-wise comparison revealed that goldfish and blunt-snout bream shared 85% identity in mtDNA sequence. Specifically, they show an 85% sequence identity in cytb as a representative of mtDNA genes (Fig. S1), and an 89% sequence identity in tfam as a representative of nuclear genes (Fig. S2). Sequence alignment allows for designing PCR primers common or specific to mtDNAs of distinct parental origins (Figs S1 and S2). The PCR primers were designed in such a way so that amplicons of different parental origins differed in size, with those common to both species being intermediate between parental and maternal (Fig. 1b). A semi-quantitative PCR analysis in serially mixed DNA samples from both species revealed a sensitivity of as low as 1‰ for detecting the blunt-snout bream mtDNA in the presence of bulk goldfish mtDNA and nuclear DNA (Fig. 1c). A similar result was obtained also for the goldfish mtDNA serially diluted in the bulk blunt-snout bream mtDNA and nuclear DNA (Fig. 1d). Therefore, reciprocal hybrids between goldfish and blunt-snout bream provide a suitable model system to quantify mtDNAs of different parental origins by sensitive PCR assays.

### Maternal inheritance and sperm delivery of mtDNA in cyprinid fish

Since MUI of mtDNA exists in the majority but not all of species examined so far, we examined the mtDNA origin in the hybrids between goldfish and blunt-snout bream. No detectable sperm mtDNA was present in the hybrid larva between female goldfish and male blunt-snout (Fig. 1e; lanes 1–3), suggesting strict MUI of mtDNA in the hybrid of cyprinid fishes. It has been reported that paternal MT undergoes uneven distribution in mouse embryos^31^, which indicates a possibility that paternal mtDNA may be present in certain adult organs. In order to test this possibility, we examined the adult organs of three germ layers from the hybrid between female goldfish and male blunt-snout. Only maternal mtDNA was detected, whereas paternal mtDNA was absent, as species-specific cytb primers generated the PCR product of merely maternal origin from goldfish but not of paternal origin from blunt-snout bream in all of the seven representative organs examined (Fig. S3). As expected, nuclear gene tfam of both maternal and paternal origins was easily detected in all of the organs (Fig. S3), which is in accordance with the hybrid identity of the organism. Thus, MUI operates in goldfish as in other cyprinid species^32^.

Two major modes operate to ensure MUI of mtDNA. One is paternal mtDNA exclusion, where sperm mitochondria do not enter into the egg but remain outside, and are thus prevented from mtDNA inheritance. This mode has been thought as exceptional because it has so far been limited to the Chinese hamster (*Cricetulus griseus*)^1^. The other is paternal mtDNA elimination, where sperm mitochondria within the intact mitochondrial sheath do enter together with the tail into the egg at fertilization but become selectively eliminated. This mode has been reported in most invertebrates and vertebrates including the fish medaka (*Oryzias latipes*)^27^. To distinguish exclusion and elimination modes, we examined the mtDNA origin in zygotes from reciprocal hybridization between goldfish and blunt-snout bream. Paternal mtDNA from either goldfish or blunt-snout bream was easily detected in the zygotes (Fig. 1e). Clearly, sperm mtDNA is delivered into the egg at fertilization but subsequently eliminated to ensure MUI in both goldfish and blunt-snout bream.

### Late elimination of paternal mtDNA in cyprinid embryos

**Figure 2:**
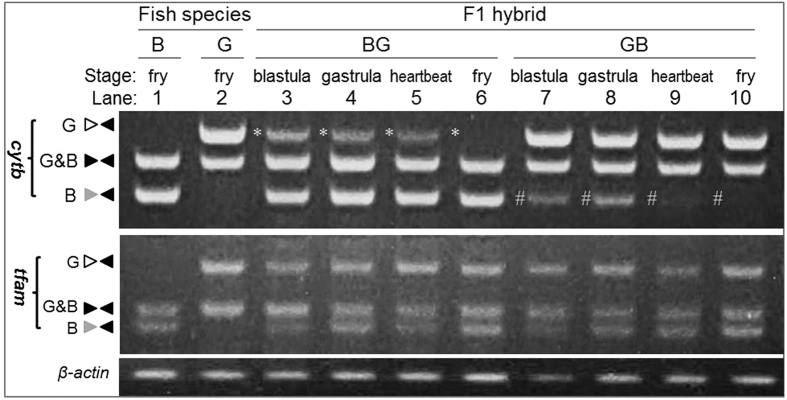
Late elimination of paternal mtDNA in cyprinid embryos. Clearly seen is persistence of sperm mtDNA in developing embryos until the heartbeat stage (24 hpf) and its disappearance in fry around hatching (34 hpf). Asterisks and hashes depict sperm mtDNA from blunt-snout bream and goldfish, respectively. DNA was isolated from 20 pooled embryos at each stage from parental species and reciprocal hybridization and analyzed by PCR at representative stages indicated. β-actin was used as a loading control. PCR and gels were run under the same conditions. Nuclear gene tfam was used for comparison. For abbreviations see legend to Fig. 1.

It is well-known that paternal mtDNA elimination takes place in early developing embryos of human and diverse animals such as mouse^33^,^34^, pig^35^, medaka^27^ and C. elegans^36^. For example, disappearance of paternal mtDNA occurs at the 4 to 8-cell transition in mouse^34^ and the 2-cell stage in medaka^27^. The fact that goldfish and blunt-snout bream make use of paternal mtDNA elimination provoked us to examine the fate of paternal mtDNA during critical stages of embryogenesis. As expected, egg mtDNA was evident throughout embryogenesis (Fig. 2). Surprisingly, sperm mtDNA was still easily detected in embryos at the blastula, gastrula and even heart-beat stages before disappearance at hatching (Fig. 2). In consistence with a hybrid nature, tfam of also paternal origin existed throughout embryogenesis (Fig. 2). Therefore, paternal mtDNA persists to advanced stages of embryogenesis and undergoes late elimination in goldfish and blunt-snout bream, which suggests that embryos until the heart beat stage in both species apparently have mitochonadrial heteroplasmy.

### Transcriptional quiescence of paternal mtDNA

**Figure 3:**
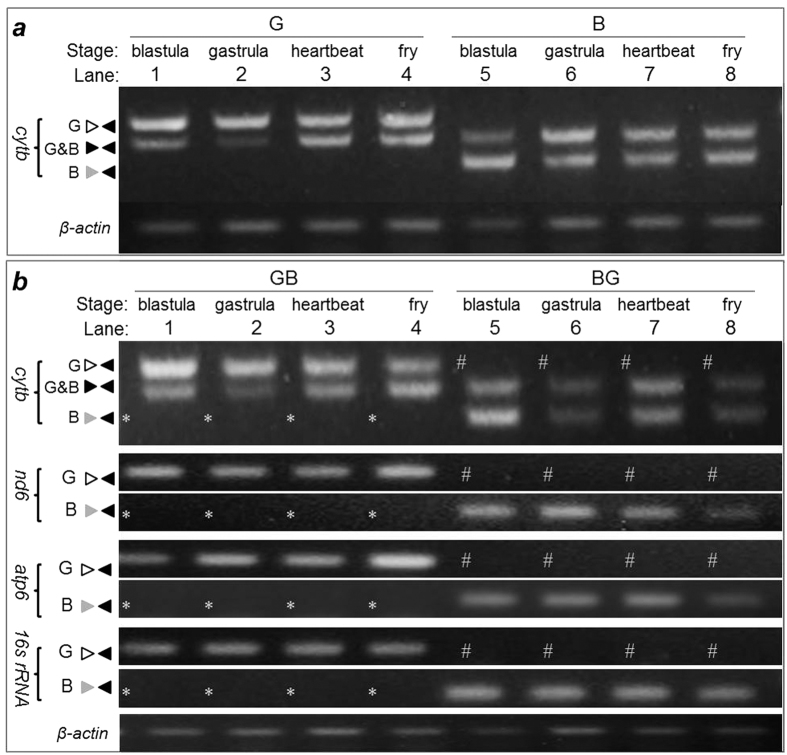
Transcriptional quiescence of paternal mtDNA. Embryos from parental species and reciprocal hybrids were analyzed by RT-PCR at representative stages indicated. β-actin was used as a loading control. (**a**) cytb expression in the embryos of goldfish (G) and blunt-snout bream (B). (**b**) Lack of gene expression from paternal mtDNA of blunt-snout bream (asterisks) and goldfish (hashes). Shown here are *cytb, nd6, atp6 and 16s rRNA* genes. For abbreviations see legend to Fig. 1.

**Figure 4:**
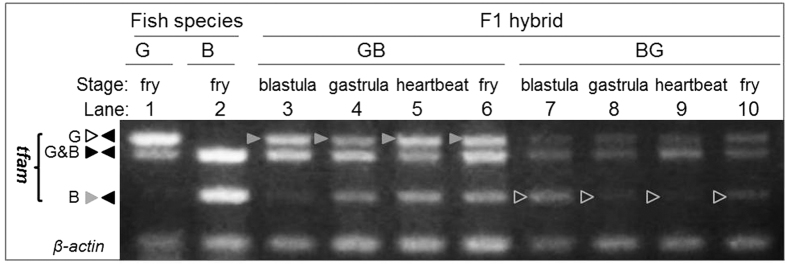
tfam expression. Both maternal (solid arrowhead) and paternal (open arrowhead) nuclear genomes show a comparable level of tfam expression. For abbreviations see legend to Fig. 1.

Delayed elimination of paternal mtDNA described above provoked us to examine the transcriptional status of maternal and paternal mtDNA at critical stages of development by using cytb and tfam as representatives of mtDNA and nuclear genes. The cytb transcript of maternal origin was easily detectable in embryos of goldfish and blunt-snout bream at stages of blastula, gastrula, heartbeat and fry (Fig. 3a). In contrast, the cytb transcript of paternal origin was never detected in reciprocal hybrid embryos at any stages examined (Fig. 3b). The lack of paternal mtDNA expression was further confirmed by three additional mtDNA genes, namely *nd6, atp6 and 16s rRNA* (Fig. 3b), For a comparison, the tfam transcript of both maternal and paternal origins was readily detected in embryos of goldfish and blunt-snout bream as well as their reciprocal hybridization (Fig. 4). Taken together, fertilization-delivered sperm mtDNA is transcriptionally quiescent throughout fish embryogenesis, which excludes any effect and phenotypic contribution by sperm mtDNA to developing embryos and thus allows for paternal mtDNA persistence and ensures MUI of mtDNA.

## Discussion

In the present study, we have performed a hybrid analysis of the germline transmission and behavior of fertilization-delivered paternal mtDNA in goldfish and blunt-snout bream as a model of cyprinid fishes. We show that MUI operates in both cyprinid fishes as in the majority of organisms examined so far^1^,^3^,^16^,^32^,^36^. Furthermore, we present two lines of evidence supporting that MUI is the consequence of paternal mtDNA elimination rather than exclusion. One is the easy detectability of paternal mtDNA in zygotes and its disappearance around the hatching stage, demonstrating the delivery of paternal mtDNA at an easily detectable level by sperm at fertilization and its subsequent elimination during embryogenesis. The other is the absence of paternal mtDNA in all of the 7 examined adult organs of three germ layers, which largely excludes the possibility that paternal mtDNA may persist in certain organs through uneven distribution. Uneven distribution of paternal mtDNA has been reported in mouse embryos as an indicator of the possible presence of paternal mtDNA in certain adult organs^31^.

A surprising observation in this study is the persistence of paternal mtDNA in developing embryos until the heartbeat stage when many major organ systems have already been established. This observation demonstrates that paternal mtDNA is eliminated late during embryogenesis in both goldfish and blunt-snout bream. This late elimination is in sharp contrast to early elimination as has been reported in all MUI organisms examined to date, including vertebrates such as the fish medaka^27^ and many mammals^33^,^34^,^35^, and invertebrates such as C. elegans^36^, where paternal mtDNA elimination occurs early in cleavage embryos. We have previously recorded recombination between maternal and paternal mtDNAs in hybrid triploids between goldfish and common carp^28^. Late elimination of paternal mtDNA revealed in this study may allow for paternal mtDNA persistence and thus favour recombination between maternal and paternal mtDNAs. Future work is needed to see whether late elimination of paternal mtDNA operates also in other animal species.

Mitochondrial heteroplasmy is usually associated with abnormal embryogenesis and diseased phenotypes in diverse organisms such as mammals^1^,^37^,^38^,^39^. Persistence of paternal mtDNA due to its late elimination indicates mitochonadrial heteroplasmy in hybrid embryos between goldfish and blunt-snout bream up to advanced stages. We have previously shown that embryos between female goldfish and male blunt-snout bream are capable of normal development as evidenced by the production of normal adult fish, whereas embryos between male goldfish and female blunt-snout bream are characterized by abnormal development and perinatal mortality^30^. These observations lead to a notion that mitochonadrial heteroplasmy has little adverse effect on embryogenesis in goldfish and blunt-snout bream and cannot be made responsible for abnormal development and perinatal death of hybrid embryos between female blunt-snout bream and male goldfish.

A striking finding obtained in this study is the transcriptional quiescence of paternal mtDNA in cyprinid embryos, which is sharp contrast to the situation of DUI mollusks where paternal mtDNA transcription occurs in both male germ cells^19^,^20^ and somatic cells^26^. This quiescence may prevent paternal mtDNA from contributing its effect and function to the cell and embryo, which in turn allows for paternal mtDNA persistence and normal development of heteroplasmic embryos as we have observed in goldfish and blunt-snout bream. In this context, transcriptional quiescence of paternal mtDNA represents a new mechanism for maternal mtDNA inheritance. Transcriptional quiescence may result from the incompatibility of mitochondrial transcription machinery and/or the inaccessibility of paternal mtDNA. In this study, we have shown that the incompatibility of mitochondrial transcription machinery is unlikely to be causative for transcriptional quiescence, because tfam RNA – whose protein product mitochondrial transcription factor A acts as a key player in mtDNA replication and transcription^40^,^41^ – does not show any difference in embryonic transcription between maternal and paternal alleles. Although work is needed to test any difference between maternal and paternal mtDNAs in transcriptional inaccessibility, our finding that paternal mtDNA is transcriptionally quiescent has important implications for treating MT-associated diseases by MT transfer or replacement as has been attempted in primates including human^12^,^13^.

## Materials and Methods

### Fish

Fish work was performed in strict accordance with the recommendations in the Guidelines for the Care and Use of Laboratory Animals of the National Advisory Committee for Laboratory Animal Research in China and approved by the Animal Care Committee of Hunan Normal University *(Permit Number: 4237).* Goldfish (red variety; *Carassius auratus*) and blunt-snout bream (*Megalobrama amblycephala*) were maintained at the National Education Ministry Breeding Center of Polyploidy Fish, Hunan Normal University as described^32^. Reproduction and reciprocal hybridization were performed by using the dry method of artificial insemination. Embryos were placed on nylon meshes in water for mass production or in Petri dishes for experimentation. Embryos in Petri dishes were regularly monitored, snap-frozen in liquid nitrogen at different stages and stored at −80 °C before use.

### Sequence analysis

Sequences were analyzed by using Blast search and aligned by using Vector NT.

### DNA and RNA extraction

DNA was extracted from freshly dissected organs of adult fish or 20 pooled embryos at each stage by using the TaKaRa MiniBEST Universal Genomic DNA Extraction Kit (TaKaRa, Japan) as described^32^. RNA was extracted by using the E.Z.N.A. Total RNA Kit II (OMEGA).

### Polymerase chain reaction

Genomic DNA PCR was run for 35 cycles (94 °C for 30 s, 58 °C for 30 s and 72 °C for 30 s) in a 25-μl volume containing 50 ng of template DNA and appropriate primers for cytb, tfam and β-actin as described^32^. Template DNA samples used were goldfish DNA, blunt-snout bream DNA or their mixtures with serial dilutions. For RT-PCR, first-strand cDNA was synthesized by using the PrimeScript^TM^ RT reagent Kit with gDNA Eraser (TaKaRa), and PCR was run for 35 cycles (94 °C for 30 s, 58 °C for 30 s and 72 °C for 30 s) in a 25-μl volume containing 10 ng of template cDNA and appropriate primers for cytb and tfam, or for 30 cycles for β-actin as a loading control. Primers used are listed in Table S1. PCR products were separated on 1.5% agarose gels and documented on the White/UV Transilluminators (UVP, Upland, CA 91786).

## Additional Information

**How to cite this article**: Wen, M. *et al*. Transcriptional quiescence of paternal mtDNA in cyprinid fish embryos. *Sci. Rep.* **6**, 28571; doi: 10.1038/srep28571 (2016).

## Supplementary Material

Supplementary Information
