# Supplementary material for: Transcriptional quiescence of paternal mtDNA in cyprinid fish embryos
Source: Sci Rep. 2016 Jun 23;6:28571. doi: 10.1038/srep28571 (PMC4917824; doi:10.1038/srep28571)
Supplement: Supplementary Information [file srep28571-s1.doc]

**Supplemental information to:**

**Transcriptional quiescence of paternal mtDNA in cyprinid fish embryos**

Ming Wen^1^*^#^, Liangyue Peng^1*^, Xinjiang Hu^1^, Yuling Zhao^1^, Shaojun Liu^1^**, Yunhan Hong^2^**

^1^State Ministry of Education Key Laboratory of Protein Chemistry and Developmental Biology, College of Life Sciences, Hunan Normal University, Changsha 410081, China; ^2^Department of Biological Sciences, National University of Singapore, Singapore 117543, Singapore

*These authors contributed equally to this work

#contains part of doctoral thesis work

**Correspondence: Professor Shaojun Liu: [lsj@hunnu.edu.cn](mailto:lsj@hunnu.edu.cn)

Key Laboratory of Protein Chemistry & Developmental Biology of State Education Ministry of China, College of Life Sciences, Hunan Normal University, Changsha 410081, China

Tel/Fax: +86-73188873074

Professor Yunhan Hong: [dbshyh@nus.edu.sg](mailto:dbshyh@nus.edu.sg)

Department of Biological Sciences

National University of Singapore

14 Science Drive 4, Singapore 117543

Tel: +65-65162915; Fax: +65-67792486

**Supplementary Table 1 | Genes and primers used for PCR analysis**

**Supplementary Figure 1 | Alignment of cytb nucleotide sequences**

**Supplementary Figure 2 | Alignment of tfam nucleotide sequences Supplementary Figure 3 | PCR analysis of mtDNA origin in adult hybrids**

**Table S1 | Genes and primers used for PCR analysis**

| Gene | Primers | | PCR product (bp) | |
| --- | --- | --- | --- | --- |
|  | Species (accession) | Name: sequence | DNA | cDNA |
| cytb | goldfish (JN105355) | cytG: ACCGCATTCTCATCCGTTAC  cytR: TTTCTACTCATCCTGCTAGTGG | 940 | 940 |
|  | blunt-snout bream (NC_010341) | cytB: AGTCCCTTATATAGGAGACACCC  cytR: TTTCTACTCATCCTGCTAGTGG | 665 | 665 |
|  | goldfish & blunt-snout bream | cytF: TC/ATACCT/ATACAAAGAAACCTG  cytR: TTTCTACTCATCCTGCTAGTGG | 806 | 806 |
| nd6 | goldfish (JN105355) | ND6G: CCCAAGCACAAATCACCAATATTC  ND6R: CCTGGGGTAGTCGTTCCGTTTTA: | 207 | 207 |
|  | blunt-snout bream (NC_010341) | ND6B: CCACGCACAAATAACCAACATCG  ND6R: TGGGGAAGTCGTTCTGTGGCT | 204 | 204 |
| atp6 | goldfish (JN105355) | ATP6G: TAACCGCCTTATTACAATTCAAGG  ATP6R: GTATATGGCAGGAGCCCTAACATG | 156 | 156 |
|  | blunt-snout bream (NC_010341) | ATP6B: CAATCGACTTATCACCCTCCAAAC  ATP6R: GAGAAGGCCTAATATATTGATGGTG | 146 | 148 |
| 16S  rRNA | goldfish (JN105355) | 16SG: CCTAGAAAAACCCACAACCTAAAT  16SR: GCTGGACCTCCTATACTTGGTTGT | 161 | 161 |
|  | blunt-snout bream (NC_010341) | 16SB: CCAAGAAAAACCCACAACTAAACA  16SR: GCTGGACCTCCTATACTCAGTTTA | 161 | 161 |
| tfam | Goldfish (KT380497) | tfamG: TCGGTGCGAATCTTCTGACG  tfamR: CTCCGAACAAGGTCTTCCCTTC | 664 | 553 |
|  | blunt-snout bream (KT380498) | tfamB: CAGACCGCTGTTATTGCAGAG  tfamR: CTCCGAACAAGGTCTTCCCTTC | 326 | 298 |
|  | goldfish & blunt-snout bream | tfamF: ACCCAAGAGACCCCTGACAG  tfamR: CTCCGAACAAGGTCTTCCCTTC | 552 | 418 |
| β-actin | goldfish & blunt-snout bream | actinF: AGAGCTATGAGCTGCCTGAC  actinR: CACTTCATGATGGAGTTGAAGG | 228 | 141 |

**Figure S1 | Alignment of cytb nucleotide sequences**. Shown are sequences and directions (arrows) of primers common to both (cytbF and cytbR) and specific to goldfish (cytbG) or blunt-snout bream (cytbB).

**Figure S2 | Alignment of tfam nucleotide sequences**. Shown are sequences and directions (arrows) of primers common to both (tfamF and tfamR) and specific to goldfish (tfamG) or blunt-snout bream (tfamB).

**Figure S3 | PCR analysis of mtDNA origin in adult hybrids.** Organs from hybrids between female goldfish and male blunt snout bream were used for DNA isolation and PCR amplification of cytb and tfam. Clearly seen is the absence of paternal cytb (asterisk) and presence of maternal plus paternal tfam in all of the hybrid organs examined. For abbreviations see legend to Figure 1.
